# Expression and localisation of c-kit and KITL in the adult human ovary

**DOI:** 10.1186/s13048-015-0159-x

**Published:** 2015-05-26

**Authors:** Astrud R Tuck, Rebecca L Robker, Robert J Norman, Wayne D Tilley, Theresa E Hickey

**Affiliations:** Robinson Research Institute, School of Paediatrics and Reproductive Health, Adelaide, South Australia Australia; Dame Roma Mitchell Cancer Research Laboratories, School of Medicine; University of Adelaide, Adelaide, South Australia Australia

**Keywords:** Human, Ovarian follicles, c-kit, KITL, Immunohistochemistry, Granulosa, Theca, PCOS

## Abstract

**Background:**

The c-kit/kit ligand (KITL) signalling axis is an essential component of ovarian folliculogenesis in mammals, but little is known about expression and localisation of its key components in the ovaries of reproductive age women. This study aimed to characterise mRNA expression of *c-kit* and *KITL* isoforms and the localisation of c-kit and KITL proteins in adult human premenopausal ovaries.

**Methods:**

This study utilised granulosa cells obtained from the preovulatory follicles of women undergoing assisted reproduction, pieces of ovarian tissue obtained from premenopausal women undergoing gynaecological surgeries and archival paraffin-embedded premenopausal ovarian tissues. Methodology included PCR for gene expression and Western blot or immunohistochemistry for protein expression.

**Results:**

Both c-kit mRNA isoforms, known as GNNK+ and GNNK-, were detected in human ovarian cortex, while KITL protein isoforms (KITL1 and KITL2) were present in ovarian cortex and human granulosa cells. Immunohistochemistry showed expression of KITL and c-kit protein in multiple cell types within follicles throughout development, from primordial follicles to large antral follicles, in addition to atretic follicles. Oocytes of all follicle stages expressed c-kit protein exclusively. Interestingly, unlike animal models, expression of both proteins displayed a less cell-type specific distribution with immunostaining present in granulosa, theca and stromal cells, suggesting that autocrine signalling occurs within the human ovary.

**Conclusion:**

The results of this study indicate that c-kit/KITL signalling also occurs in the human ovary, as established in various animal models, and may involve previously unknown autocrine signalling.

**Electronic supplementary material:**

The online version of this article (doi:10.1186/s13048-015-0159-x) contains supplementary material, which is available to authorized users.

## Background

KITL is a cytokine growth factor secreted by granulosa cells of ovarian follicles that exerts intra-follicular paracrine signals via stimulation of the c-kit receptor, which is expressed in theca cells and oocytes [1–3]. Transgenic mouse models have been utilised to demonstrate critical roles for the key ligand and receptor components of the c-kit/KITL signalling pathway during folliculogenesis ([4–8]; 2004). Expression and function of these key components have also been elucidated in other animal models including rat, rabbit, chicken, goat, pig and cow [1, 2, 9–11], and these have demonstrated several key roles that are essential for the maintenance of ovarian function and fertility. These roles include primordial follicle activation, promotion of primary to secondary follicle transition, preantral follicle growth, formation of the theca layer, thecal steroidogenesis and anti-apoptosis [3, 9].

Few studies have examined the expression and function of c-kit and KITL in human ovarian tissues. Evidence suggests that while c-kit/KITL signalling does not appear to be involved in human primordial follicle activation [12], it may have roles in follicle survival and thecal steroidogenesis similar to animal models. Currently, consensus is lacking over the cell type distribution of these two signalling proteins in human ovarian tissues [12–14], which hampers understanding of their role in human ovarian function. Differences among studies may be largely due to the detection methodologies employed and the difficulty of obtaining human ovarian tissues that contain sufficient material to make conclusive observations. In one study, expression of KITL was found to be detected only in the oocytes of fetal and adult ovaries [14], a finding which is in stark contrast to animal studies where KITL protein is localised exclusively to granulosa cells. For c-kit expression, one study demonstrated the presence of c-kit protein on the membranes and cytoplasm of preantral granulosa cells of adult human ovaries [12], while another study reported the presence of c-kit protein only in the oocytes of preantral follicles [14]. These data that confine c-kit expression to the preantral follicle cohort are not consistent with the detection of soluble c-kit protein in the follicular fluid of antral follicles [13]. The latter finding suggests that the theca layer secretes soluble c-kit into the follicular fluid, although this remains to be confirmed. Inconsistencies in previous studies involving human ovarian tissues highlight the need for a more comprehensive study into the expression of the c-kit/KITL signalling pathway to reveal potential species differences.

Polycystic ovary syndrome (PCOS) is a common endocrine disorder associated with infertility and metabolic morbidities [15, 16]. Polycystic ovaries (PCO) are significantly enlarged, fibrotic, and contain an increased number of antral follicles in a state of developmental arrest. Although the specific roles of c-kit and KITL in the human ovary have not been established, based on their established roles in animal models perturbed KITL signalling could feasibly contribute to multiple phenotypic characteristics of PCO such as abnormal oocyte growth, increased follicle and stromal density, thecal hypertrophy, and increased thecal cell androgen biosynthesis [15, 17–22]. To date, the c-kit/KITL signalling system has not been examined in relation to PCO or PCOS, in part because it is extremely difficult to obtain normal pre-menopausal ovarian tissues for comparison.

Numerous isoforms of KITL and c-kit have been described in the tissues of various animal species. In terms of KITL, most species, including humans, express two isoforms (KITL1 and KITL2) that arise via alternative mRNA splicing of the *KITL* gene. The chicken is uniquely reported to express six KITL isoforms in the ovary [23]. Two c-kit isoforms, known as GNNK+ and GNNK-, also arise by mRNA splicing and differ only by the presence or absence of four amino acids (GNNK) in the translated protein. These c-kit isoforms have been detected in the bovine ovary, but their expression in the reproductive tissues of other species remains unexplored. Animal studies have demonstrated the presence of KITL isoforms within the ovary, and mouse models have indicated the essential role of KITL2 for oocyte growth and survival [24]. To date, no study has ever comprehensively examined KITL or c-kit isoform expression in the human ovary.

To address current gaps in knowledge about the KITL/c-kit signalling pathway in the ovaries of reproductive age women, the specific aims for this study were to characterise the mRNA expression of c-kit and KITL isoforms and to localise c-kit and KITL protein in adult premenopausal human ovarian tissues, including ovarian tissues from women clinically diagnosed PCOS. This study demonstrates the presence of the KITL and c-kit protein throughout folliculogenesis in a less cell-type specific distribution than animal follicles. Furthermore, this study shows for the first time that KITL and c-kit isoforms are expressed in human ovarian tissue.

## Materials and methods

### Human ovarian tissue collection

#### Archival ovarian tissue

Formalin-fixed, paraffin-embedded ovarian tissues were obtained from hospital archives originally owned and operated by the Institute of Medical and Veterinary Science (IMVS; Adelaide, South Australia) under ethics approvals from the Royal Adelaide Hospital, the Central Northern Adelaide Health Service, and the University of Adelaide. Records corresponding to premenopausal women who had surgery for benign gynaecological conditions were collated and the associated tissue blocks were retrieved where possible. Fourteen tissues were collected and histologically assessed by a pathologist. Of these, six had histologically normal morphology and eight had morphology characteristic of polycystic ovaries (PCO), two of which had diagnoses of PCOS in the clinical notes. The mean age of non-PCOS patients was 35.5 years, while the mean age of PCOS patients was 37 years. Due to the limited information contained in the clinical notes of archival tissue, BMI was not available.

Within ovarian tissues, follicle classifications were performed as follows: a follicle was classified as primordial if it consisted of an oocyte surrounded by a single layer of flattened granulosa cells, and classified as primary if the oocyte was surrounded by a single layer of cuboidal granulosa cells. A secondary follicle had two or more layers of granulosa cells without an antrum, and an antral follicle had a large cavity, several layers of granulosa cells and a defined basal lamina and thecal layer. Large antral atretic follicles were classified by having an oocyte with an irregular membrane or a thinning layer of granulosa cells with pyknotic nuclei. Stages of atresia were assessed by the level of degradation of the granulosa cell layer and the appearance and definition of the basal lamina and theca layer. Germinal inclusion cysts were classified as fluid-filled cavities of various sizes situated in the cortex, surrounded by one or more layers of cuboidal or rectangular epithelial cells.

#### Fresh ovarian tissue

Fresh pieces of human ovarian cortex approximately 1 cm^3^ in size were obtained from consenting premenopausal (*n* = 3) women having oophorectomy as part of gynaecological surgery at the Royal Adelaide Hospital, South Australia, Australia. The study was approved by the ethics committees of the Royal Adelaide Hospital and the University of Adelaide. Tissue was immediately snap frozen upon collection and stored in liquid nitrogen.

#### Human granulosa cells

Following informed consent, mural granulosa cells (MGC) and cumulus granulosa cells (CGC) from pre-ovulatory follicles were collected from women (*n* = 4) who were receiving assisted reproduction at Repromed (Dulwich, South Australia). The study was approved by the ethics committees of the Women’s and Children’s Hospital, the University of Adelaide, and Repromed. All women underwent a standard hyperstimulation protocol with gonadotropin-releasing hormone (GnRH) down-regulation and subsequent gonadotrophin stimulation prior to oocyte retrieval. Cumulus-oocyte complexes were collected by transvaginal aspiration. MGCs were isolated by centrifugation of follicular fluid, mixed with PBS and layered onto onto a 50:50 *v/v* mixture of Percoll (Amersham Biosciences, Australia). After centrifugation to produce a density gradient, MGCs were removed and subjected to a second density gradient to remove any remaining blood contamination. Cells were washed in PBS and then pooled from all follicular aspirates representing one patient. Cumulus granulosa cells were removed from the oocyte by manually trimming (*in vitro* fertilisation) or hyaluronidase treatment (intracytoplasmic sperm injection). All cells were snap frozen and stored at −80 °C.

### RNA isolation

Total RNA was extracted from cells using RNeasy Micro Kit (Qiagen, Hilden, Germany) with on-column DNase treatment (RNase-Free DNase Set, Qiagen) and RNA yield was assessed by spectrophotometer (ND-1000; NanoDrop Technologies Inc, Wilmington, DE, USA). Reverse transcription was performed on 100 ng of total RNA using the iScript First-Strand cDNA Synthesis Kit (Bio-Rad Laboratories, NSW, Australia).

### Polymerase chain reaction

PCR to detect c-kit isoforms was performed using a primer pair designed to show both isoforms as previously described [25]: (forward: 5′-GGGGGATCCGATGTGGGCAAGACTTCT-3′; reverse 5′-CAGCAAAGGAGTGAACAG-3′). PCR was performed using a Pfu polymerase (Stratagene, La Jolla, CA, USA) and thermocycler conditions as follows: 94 °C for 7 min, then 25 cycles consisting of 94 °C for 1 min, 45 °C for 1 min and 72 °C for 12 min. PCR products were visualised on a 4 % agarose gel to detect products 93 and 81 bp in size (GNNK+ and GNNK–respectively). cDNA from the human leukaemic cell line, K562, was obtained from the Leukaemia Unit, Department of Molecular Pathology (SA Pathology, Adelaide, South Australia, Australia) and used as positive control for c-kit expression [26, 27].

### Western blot

Human breast tissue was obtained from the Dame Roma Mitchell Cancer Research Laboratories (University of Adelaide, South Australia) for use as a positive control for c-kit immunostaining. Frozen ovarian cortex or frozen breast tissue, approximately 1 cm^3^, was ground to a fine powder using a mortar and pestle. Liquid nitrogen ensured that samples remained frozen at all times. Breast, ovarian cortex, MGC and CGC were lysed using RIPA buffer including protease inhibitor cocktail tablets, followed by centrifugation at 10,000 rpm for 10 min at 4 °C. Concentration of protein was determined using a Bradford protein assay at 595 nM. CCRF-HSB-2, a human leukaemic cell lysate, was commercially obtained for use as a positive control for c-kit (Santa Cruz Biotechnology, Santa Cruz, CA, USA) as previously demonstrated [28] and used according to the supplier’s instructions.

Protein samples were prepared by adding 6x loading dye, denaturing at 100 °C for 5 min then centrifuging for 1 min. Proteins (20 μg) were electrophoresed on Criterion XT precast gels in 1x XT MOPS running buffer at 150 V for a total running time of 60–90 min. Precision Plus Protein Standards Dual Colour molecular weight marker was run alongside protein samples. Proteins were transferred to a Hybond-C nitrocellulose membrane under wet transfer conditions, using a Criterion Blotter according to the manufacturer’s instructions. Briefly, the transfer was set up in cold 1x transfer buffer with the gel and membrane stacked between Whatman filter paper, and transferred in cold 1x transfer buffer at 400 mA for 60–90 min. Protein transfer was checked by staining the membrane for 1 min in Ponceau stain, followed by brief rinsing in TBST. Membranes were blocked in TBST containing 3 % skim milk powder for 1 h, followed by incubation with either the c-kit primary antibody (1:1000, polyclonal rabbit anti-human CD117, DakoCytomation, Glostrup, Denmark) or the KITL primary antibody (1:1000; anti-stem cell factor, Millipore, Billerica, MA, USA) overnight at 4 °C. The appropriate HRP-conjugated secondary antibody (1:2000) was added for 30 min. Each primary and secondary antibody was diluted in TBST containing 1 % skim milk powder. Membranes were washed 3 times for 10 min in TBST following incubation with each antibody. All steps were performed with gently rotation at room temperature, except for overnight incubations which were performed at 4 °C. Bound antibody was visualised using SuperSignal West Dura Extended Duration Substrate, according to the manufacturer’s instructions, and exposed to Amersham Hyperfilm ECL. The film was developed in a dark room using standard developing and fixative reagents. Densitometry analysis was performed by measuring band intensity using the AlphaImager 2200 gel documentation system (Alpha Innotech, San Leandro, CA, USA).

### Immunohistochemistry

**Fig. 1:**
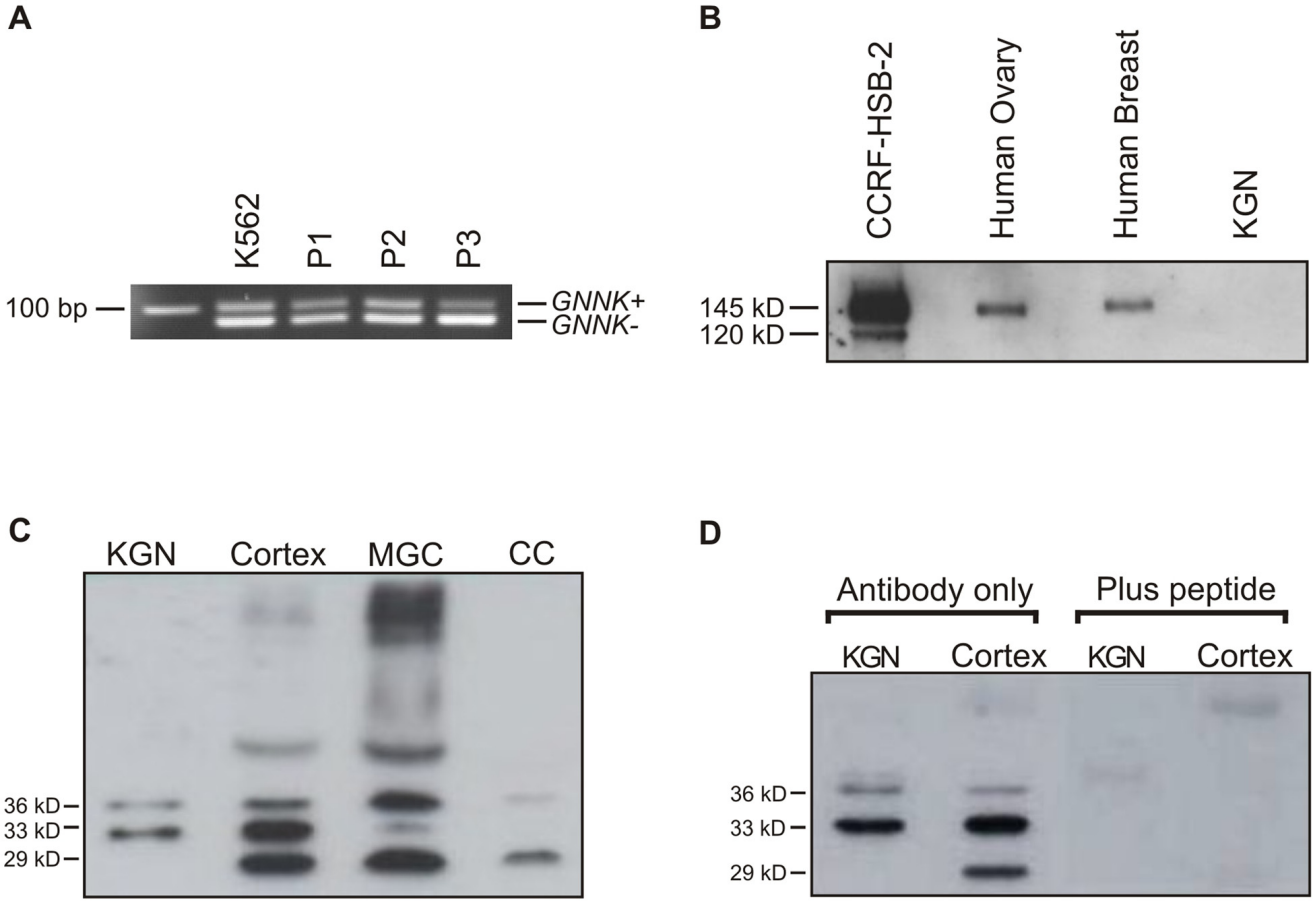
c-kit and KITL isoforms are expressed in human ovary **(a)** c-kit isoforms, *GNNK+* and *GNNK*−, were both present in ovarian cortex from three premenopausal women. Human leukaemia cell line K562 which expresses both isoforms was used as a positive control. **(b)** Western blot depicting a single 145 kDa band in human ovary and breast tissue lysates. CCRF-HSB-2 was used as a positive control and contains the 145kD band, representing glycosylated protein, as well as a smaller, 120kD, band representing non-glycosylated c-kit. **(c)** KITL protein isoform expression in the KGN human granulosa tumour cell line as a positive control, mural granulosa cells (MGC), cumulus cells (CC) and ovarian cortex containing some preantral follicles. Specific bands for KITL1 (36kD) and KITL2 (33kD) are present in all samples. An unknown 29kD band appears in the primary tissues but is absent in KGN cells. **(d)** Overnight incubation of the KITL antibody with its specific peptide demonstrates specificity

A range of c-kit antibodies were tested and optimised using human ovarian tissue and human gastrointestinal stromal tumour (GIST) as a positive control (obtained from IMVS, Adelaide). From these, CD117 rabbit polyclonal antibody (DakoCytomation) was chosen as it was the antibody that provided a pattern of staining in GIST most similar to that used for diagnosis as shown in previous studies [29, 30], and had low background staining in ovarian test sections. This antibody has also been used in a previous study examining c-kit expression via immunohistochemistry in adult human ovaries [31]. No blocking peptide was available to confirm antibody specificity, but the presence of a single band on Western blot indicates the antibody is specific (Fig. 1b). Similarly, a range of KITL antibodies were tested and optimised using human breast carcinoma as a positive control (obtained from Dame Roma Cancer Research Laboratories, Adelaide). The antibody (Cell Signalling Technology, Inc., MA, USA) used in this study detects both isoforms as it was raised against a peptide sequence in the amino terminus of human KITL that is common to both isoforms, and this was confirmed in human breast carcinoma (data not shown). Antibody specificity in human ovarian tissue and breast carcinoma was confirmed by pre-absorption with the antigenic peptide (produced by Auspep, VIC, Australia, from the KITL sequence provided by Cell Signalling Technology), which resulted in a complete absence of staining in all tissue areas.

Immunohistochemistry was performed on tissue sections (3 μm) as previously described [32], except antigen retrieval in 10 mM citrate buffer (pH 6.5) was performed using a Decloaking Chamber (Biocare Medical, Concord, CA, USA) following the manufacturer’s protocol. Quenching of endogenous peroxidise activity (6 % H_2_O_2_ in methanol for 5 min at room temperature) was performed followed by blocking of non-specific antigens with 5 % goat serum in PBS for 1 h at room temperature. Tissues were stained with a 1:300 dilution of c-kit (CD117) (DakoCytomation, Glostrup, Denmark) or 1:100 dilution of KITL antibody (Cell Signalling Technology, Inc., MA, USA) overnight at 4 °C. A different KITL primary antibody to the one used for Western blot was utilised for immunohistochemistry, due to the Western blot antibody being unsuitable for use in immunohistochemistry. Visualisation of immunoreactivity was performed using a standard immunoperoxidase reaction. Biotinylated anti-rabbit antibody (1:400), streptavidin-HRP complex (1:500) and diaminobenzidine tetrahydrochloride was used to generate an insoluble brown deposit. Stained sections were scanned using the NanoZoomer image system (Hamamatsu, Japan) set at a magnification of 40X.

## Results

### c-kit mRNA and protein expression in human ovarian cortex

To examine the presence of *GNNK c-kit* mRNA isoforms, RT-PCR was performed on mRNA isolated from ovarian cortical tissue collected from pre-menopausal women (*n* = 3) between 40 and 50 years of age. Haematoxylin and eosin staining was performed in corresponding tissue sections, which revealed that several healthy preantral follicles were present within all the tissues (data not shown). Figure 1a shows the presence of two distinct PCR products in ovarian cortical tissue from three different patients as well as in K562 human leukaemia cells, which correspond to the *GNNK+* (93 bp) and *GNNK−* (81 bp) isoforms. Although not quantitative, these results indicate that human ovarian cortical tissue expresses both mRNA isoforms of c-kit.

To examine the presence of c-kit protein, ovarian cortical tissue lysates were assessed by Western blot. Human leukaemic cell line lysate, CCRF-HSB-2, and human breast tissue lysate were used as positive controls since both have previously been shown to express c-kit [28, 33]. As expected, CCRF-HSB-2 cells possessed c-kit protein bands of 145 kDa and 120 kDa, thought to be glycosylated and unglycosylated forms of c-kit, respectively [34, 35]. Note that the GNNK+ and GNNK- isoforms cannot be distinguished via Western Blot due to the very small size difference between their corresponding proteins. Ovarian cortex and breast tissue lysates contained a single 145 kDa band (Fig. 1b), suggesting that unglycosylated forms of c-kit are not present. The KGN ovarian granulosa tumour cell line was added as a potential negative control for c-kit protein, as we were unable to detect mRNA for either c-kit isoform in this cell line (data not shown).

### KITL protein isoform expression

To examine KITL protein isoform expression in samples representative of different stages of follicle development, Western blot was performed using lysates from: 1) KGN cells that have a phenotype similar to granulosa cells from preantral/ early antral follicles [36]; 2) human pre-menopausal ovarian cortex containing primordial and preantral follicles; 3) mural granulosa cells (MGC) aspirated from the pre-ovulatory follicles of a women receiving assisted reproduction and 4) cumulus granulosa cells (CGC) stripped from the cumulus-oocyte-complexes aspirated from the pre-ovulatory follicles of the same woman. KGN cells contained both KITL1 and KITL2 protein isoforms, detected as 36 kDa and 33 kDa bands as expected. Lysates derived from primary tissues also expressed both KITL isoforms to different degrees but an additional 29 kDa band was also consistently present (Fig. 1c). The Western blot shown is representative of three independent experiments. These qualitative findings were reproducible when analysing samples derived from other patients. Due to the nature of the samples, which represented either a whole tissue lysate or distinct enriched populations of cells, common loading control markers were not appropriate use and quantitative comparisons could not be made among the different samples. However, in general, levels of KITL2 (33 kDa) appeared proportionally higher than levels of KITL1 in KGN cells and ovarian cortical tissue, while levels of KITL1 appeared proportionally greater than KITL2 in MGC and CGC lysates. To further validate the specificity of each band, the KITL primary antibody was incubated overnight with a KITL peptide (Auspep, West Melbourne, VIC, Australia). The 29 kDa band was abolished along with the two expected KITL isoforms bands following peptide competition (Fig. 1d), suggesting that it is also an isoform of KITL, albeit one that is larger than the secreted form of KITL which is known to be approximately 18 kDa in size [9]. Interestingly, Ensembl Human (http://asia.ensembl.org/Homo_sapiens/Transcript/Summary?db=core;g=ENSG00000049130;r=12:88492793-88534762;t=ENST00000378535) includes a transcript of a third *KITL* splice variant which encodes a protein of similar size to the 29 kDa band, suggesting that this protein may be a third isoform. Collectively, these observations suggest that KITL2 is the predominant isoform expressed during earlier stages of follicle development, while KITL1 is predominantly expressed in the later, preovulatory, stage.

### c-kit and KITL expression and localisation throughout follicle development

To further localise c-kit and KITL protein expression, human ovarian sections were subjected to immunohistochemistry. The patterns of staining intensities observed in preantral and antral follicle types are summarised in Table 1. A human gastrointestinal stromal tumour (GIST) was used as a positive control for c-kit immunostaining (Additional file 1: Figure S1) as this antibody is routinely used for diagnosis of GIST [29, 30]. Human breast tissue was used as a positive control for KITL immunostaining (Additional file 2: Figure S2) and peptide competitions via incubation of the KITL primary antibody with its specific peptide abolished immunoreactivity in both breast and ovarian tissue (Additional file 2: Figure S2).

**Table 1:** Summary of the patterns of c-kit and KITL staining intensities observed in preantral and antral follicles

	Primordial		Primary			Antral	
	OOC	GC	OOC	GC	OOC	GC	TH
c-kit	++/+++	─/+	++/+++	─/+	n/a	+	+
KITL	─/+	─/+	─/+	─/+	n/a	+/++	+/++

- negative; + weak; ++ moderate; +++ strong; n/a none available

OOC, oocyte; GC, granulosa cells; TH; theca layer

**Fig. 2:**
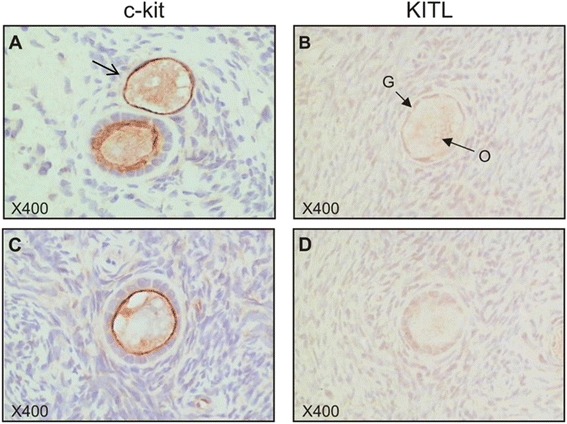
c-kit and KITL are present in preantral follicles. Representative images of c-kit **(a, c)** and KITL **(b, d)** immunostaining in primordial **(a, b)** and primary **(c, d)** follicles. O: oocyte; G: granulosa cells. The open arrow indicates a primordial follicle

In primordial and primary follicles, c-kit immunostaining was present on the oolemma and also present in the ooplasm of the majority of oocytes (Fig. 2a, c). Some follicles also showed diffuse cytoplasmic staining in all granulosa cells surrounding the oocyte. Little to no staining was observed in the stromal cells surrounding follicles. KITL immunostaining was weak or absent in the ooplasm of primordial and primary follicles and in the cytoplasm of their associated GC (Fig. 2b, d). Sporadic, weak immunostaining was also observed in the stroma surrounding these follicles.

**Fig. 3:**
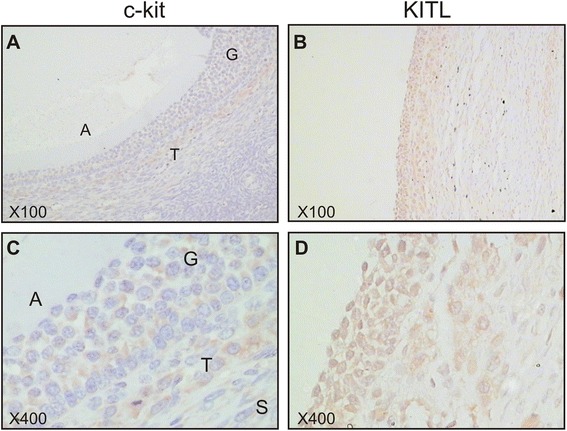
c-kit and KITL are present in antral follicles. Representative images of c-kit **(a, c)** and KITL **(b, d)** immunostaining at low **(a, b)** and high **(c, d)** magnifications. A: antrum; G: granulosa cells; T: theca layer; S: stroma

Antral follicles showed diffuse, weak cytoplasmic c-kit staining within granulosa cells and the thecal layer (Fig. 3a, c). Moderate cytoplasmic KITL staining was present within all granulosa cells and the thecal layer of antral follicles (Fig. 3b, d). Weak KITL immunostaining was present in adjacent stroma. There were no oocytes present in any of the antral follicles examined.

**Fig. 4:**
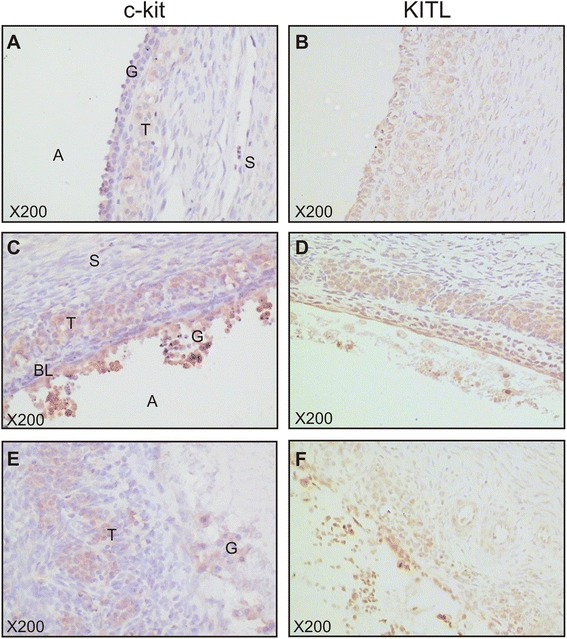
c-kit and KITL are expressed during various stages of atresia. **(a, b)** Intact granulosa layer, theca layer and basal lamina. **(c, d)** Disassociating granulosa cell layer with pyknotic nuclei. **(e, f)** Atretic follicle with little remaining of the granulosa layer and no distinction between the basal lamina and theca layer. A: antrum; G: granulosa cells; BL: basal lamina; T: theca layer; S: stroma

Immunostaining was also examined in atretic follicles. A follicle in the early stages of atresia with an intact granulosa layer, basal lamina and theca layer showed diffuse cytoplasmic c-kit and KITL staining in the granulosa and theca cells (Fig. 4a, b). In a follicle with a degrading granulosa layer, c-kit and KITL staining remained present in the cytoplasm of apoptotic granulosa cells and theca cells (Fig. 4c, d). KITL and c-kit immunostaining was present in theca cells and the few remaining granulosa cells of a follicle with advanced atresia lacking distinction between the areas of remaining granulosa cells, basal lamina and theca layer (Fig. 4e, f).

### c-kit and KITL expression and localisation in ovarian follicles of women with PCOS

**Fig. 5:**
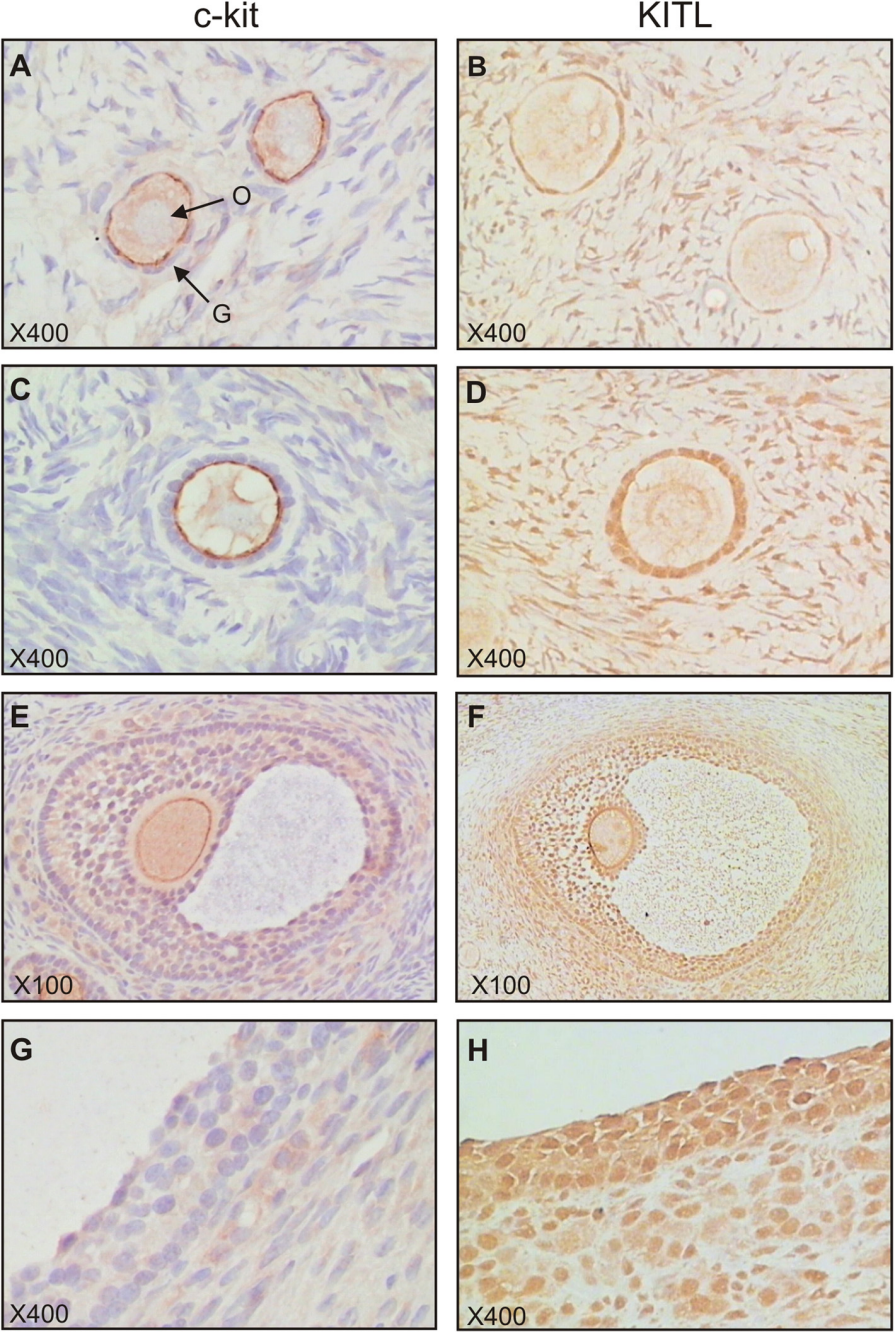
c-kit and KITL in PCOS preantral and antral follicles. Representative images of primordial follicles **(a, b)**, primary follicles **(c, d)**, secondary follicles **(e, f)** and antral follicles **(g, h)**. O: oocyte; G: granulosa cells

The cohort of ovarian tissues collected included two patients clinically diagnosed with PCOS. Interestingly, KITL staining was markedly more intense in granulosa cells, oocytes and surrounding stroma of follicles at all stages of development (Fig. 5) compared to follicles in non-PCOS ovaries (Figs. 2 and 3). While there were no secondary follicles present in non-PCOS ovaries for comparison, KITL staining was intense within the oocyte, granulosa cells and surrounding stroma cells of a secondary follicle present within one of the PCOS tissues (Fig. 5e, f). While the intensity of c-kit staining appeared no different in the two PCOS ovaries compared to non-PCOS, it was observed that oocytes in preantral follicles of PCOS patients more consistently exhibited strong oolemma staining. Moderate c-kit staining was present within the oolemma of the secondary follicle, and diffuse cytoplasmic staining was also present in granulosa cells and surrounding stroma cells (Fig. 5e, f). Granulosa cells of PCOS preantral follicles appeared to have mostly negative c-kit staining. C-kit immunostaining did not appear visually different in PCOS antral follicles compared to non-PCOS. Although this represents a small set of observations, the results are suggestive of an aberrant KITL/c-kit signalling axis in PCOS.

## Discussion

This study demonstrates that the c-kit/KITL signalling pathway is expressed at multiple stages of follicular development in adult, reproductive age human ovaries. Moreover, this is the first study to document the presence of specific c-kit and KITL isoforms in human ovarian tissue. Their presence in preantral and antral follicle stages is indicative of their involvement in human folliculogenesis.

Unlike mouse ovaries, in which c-kit and KITL are expressed in distinct cellular compartments and signal exclusively in a paracrine manner, the expression pattern of c-kit and KITL in human ovaries displayed a less cell-type specific distribution. Human granulosa, theca and stromal cells all displayed at least some weak staining for both proteins, suggesting that autocrine signalling occurs in these cells types. The presence of c-kit protein in adult granulosa cells is supported by previous studies, which demonstrated the presence of *c-kit* mRNA and protein in pregranulosa cells and granulosa cells of primordial follicles in human fetal ovaries [12, 14, 37]. This finding suggests that the roles of KITL previously established in animal models may also be present in the human ovary, and furthermore, that these functions are perhaps regulated in a differential manner (e.g., autocrine versus paracrine). Given that KITL promotes preantral follicle growth and oocyte development [1, 38–40], human preantral follicles that are positive for granulosa cell c-kit may possess a selective advantage over follicles that do not express the KITL receptor.

c-kit and KITL protein was also found to be co-expressed in the theca layer of all antral follicles. This suggests that the roles of KITL in formation and function of the theca layer, as shown in the bovine ovary [41–43], may remain conserved in the human ovary. In contrast, a study examining c-kit immunostaining in human adult ovaries did not show any c-kit protein in the theca layer [12]. This is most likely due to the primary antibody used, ACK-2, which has been utilised primarily to inhibit c-kit in in vivo mechanistic studies rather than for immunohistochemistry. The antibody used in our study was carefully selected from a range of antibodies that were extensively tested, and has also been established in studies to be the most optimal and accurate antibody for use in diagnosis of GIST [29, 30]. No blocking peptide was available, but the presence of a single band on Western blot indicates the antibody is specific.

This study demonstrated that both KITL and c-kit are present in granulosa and thecal cells of antral follicles throughout atresia until cell layers have undergone total degradation. In sheep, KITL gene expression has been shown to be maintained in the granulosa cells of atretic follicles [44], while in mice, KITL expression disappears [5]. This is the first study to demonstrate the presence of c-kit expression in atretic follicles, and it is unclear whether the KITL/c-kit system plays an active role in dying follicles.

This study is the first to demonstrate KITL1 and KITL2 protein expression in human granulosa cell types. Interestingly, KGN cells, which are representative of preantral follicles, showed evidence of higher levels of KITL2 which may suggest a greater role for KITL during early follicle development. This isoform has been demonstrated to be crucial for fertility and normal folliculogenesis in mice [7, 45], in addition to mouse oocyte growth and survival *in vitro* [24]. The anchorage of KITL2 in the cell membrane is believed to be responsible for its prolonged receptor activation compared to KITL1, suggesting that KITL2 signalling may be more efficient and promotes sustained effects [46]. In our study, granulosa cells from preovulatory follicles showed higher levels of KITL1, which is believed to initiate shorter-term, transient effects due to faster receptor internalisation and degradation of the ligand/receptor complex [46]. Many established roles for KITL occur during earlier follicle development [9]. The presence of increased levels of KITL2 in preantral granulosa cells may suggest that KITL plays a greater or more prolonged role during early human folliculogenesis.

We also report for the first time that both *GNNK+* and *GNNK−* mRNA isoforms of c-kit are present within the human premenopausal ovary, but it remains an important unanswered question which individual cell types specifically express each isoforms. In NIH3T3 fibroblast cells, *GNNK−* causes faster receptor phosphorylation and internalisation, and more extensive activation of the MAPK pathway than *GNNK+* [25]. The presence of each isoform in the human ovary may suggest that regulation of KITL signalling occurs at the receptor level, and is determined by the particular c-kit isoform being expressed. This may have important implications in ovarian pathologies where perturbed c-kit/KITL signalling may play a role, such as PCOS which is mentioned further below. This observation, if confirmed in individual cell types, may provide some knowledge of the mechanisms of KITL signalling in the human ovary via activation of specific c-kit isoforms.

A limitation of our study that should be noted was the inability to quantify the levels of c-kit cDNA and KITL protein for comparison in each cell and tissue type. As these experiments were performed on whole tissue lysates and distinct, isolated cell types, a housekeeping gene or loading control marker could not be assumed to have consistent expression across each tissue type. Furthermore, we did not have the required numbers of participants to perform rigorous statistical analysis of expression levels. Thus, our study demonstrates only the presence of c-kit and KITL isoforms in ovarian tissues and cell types and is not able make quantitative conclusions on their expression levels.

Interestingly, this study observed markedly increased KITL immunostaining intensity in follicles at all stages of development within the two PCOS ovaries that were examined. Diffuse staining was also present within the oocytes of all preantral follicles, unlike the follicles of the morphologically normal ovaries examined in this study, suggesting altered expression of KITL in PCOS oocytes. It could be postulated that increased KITL levels in PCOS ovaries may underlie several of the abnormalities observed in PCOS, such as increased ovarian reserve [2, 47–50] or enlarged oocytes [51], due to the diverse roles KITL has in animal models. Further study is required to confirm whether KITL protein is increased in PCOS using a larger sample size with ovarian tissues corresponding to women with more extensive clinical documentation for PCOS, including relative ovulation frequency.

In conclusion, this study has demonstrated the presence of KITL and c-kit in the adult human ovary throughout follicle development, in addition to showing the presence of each isoform. This suggests that the KITL/c-kit system is involved in human folliculogenesis, and further study is required to elucidate their roles and importance in human follicle development and fertility.

## Additional files

Additional file 1: Figure S1. c-kit immunostaining in human gastrointestinal stromal tumour (GIST) used as a positive control. (A, B) Cytoplasmic and membrane staining present in distinct areas. (C) Negative control consisted of omission of the primary antibody. Arrows indicate cells with membrane staining.

Additional file 2: Figure S2. KITL immunostaining in human breast and ovarian tissue. Human breast carcinoma (A-C) and human ovarian cortex tissue sections (D-F) used as controls for KL immunostaining. (A, D) Positive KITL staining. (B, E) Negative control which consisted of omission of the primary antibody. (C, F) Confirmation of KITL antibody specificity by incubation of the primary antibody overnight with its specific peptide prior to immunostaining.

## Abbreviations

KITL: Kit ligand
CGC: Cumulus granulosa cells
MGC: Mural granulosa cells
GIST: Gastrointestinal stromal tumours
PCOS: Polycystic ovary syndrome
