# Supplementary figures and images for: Expression and localisation of c-kit and KITL in the adult human ovary
Source: J Ovarian Res. 2015 May 26;8:31. doi: 10.1186/s13048-015-0159-x (PMC4460643; doi:10.1186/s13048-015-0159-x)

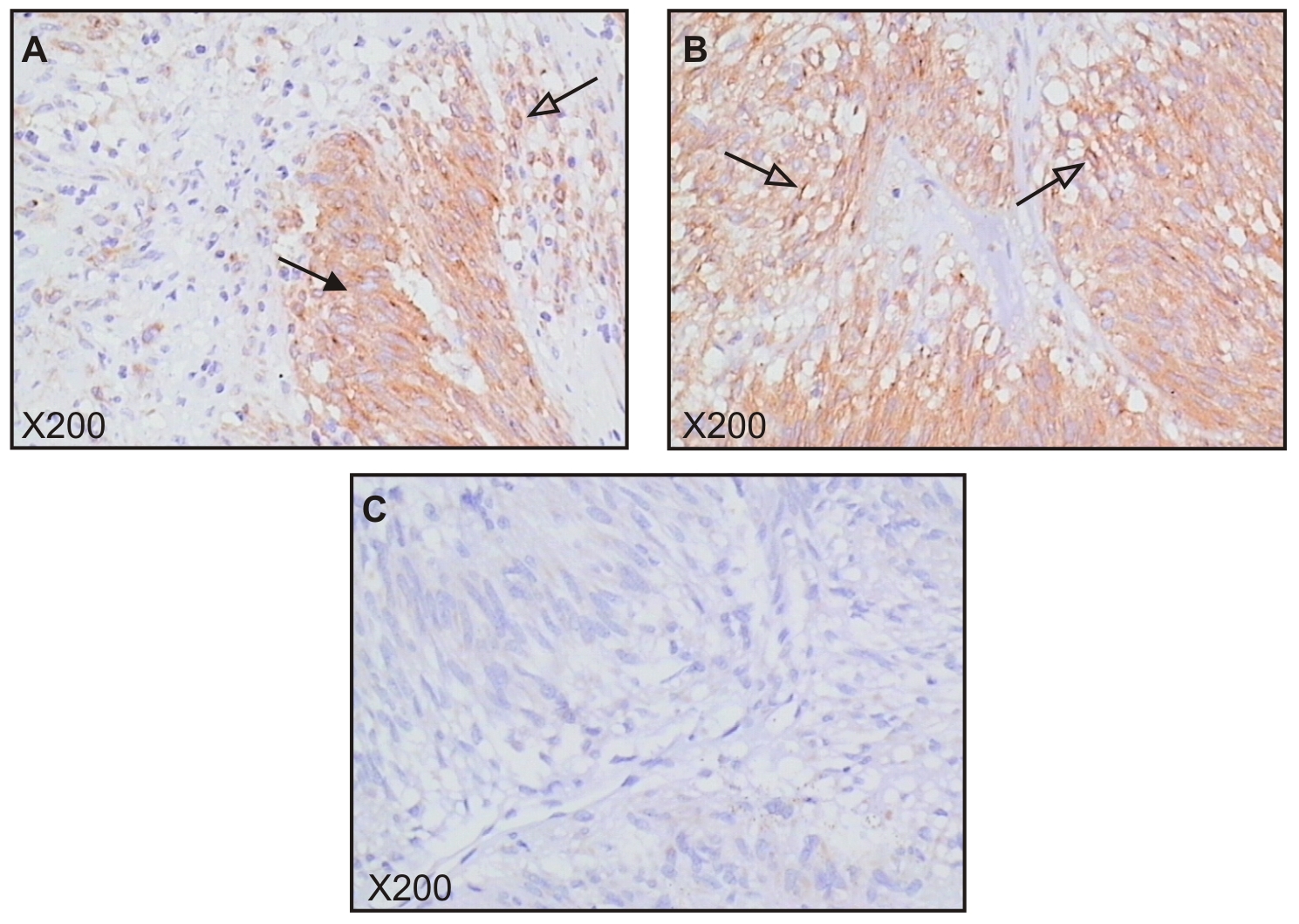

Supplement: Supplementary file 1 — c-kit immunostaining in human gastrointestinal stromal tumour (GIST) used as a positive control. (A, B) Cytoplasmic and membrane staining present in distinct areas. (C) Negative control consisted of omission of the primary antibody. Arrows indicate cells with membrane staining. [file 13048_2015_159_MOESM1_ESM.jpeg]

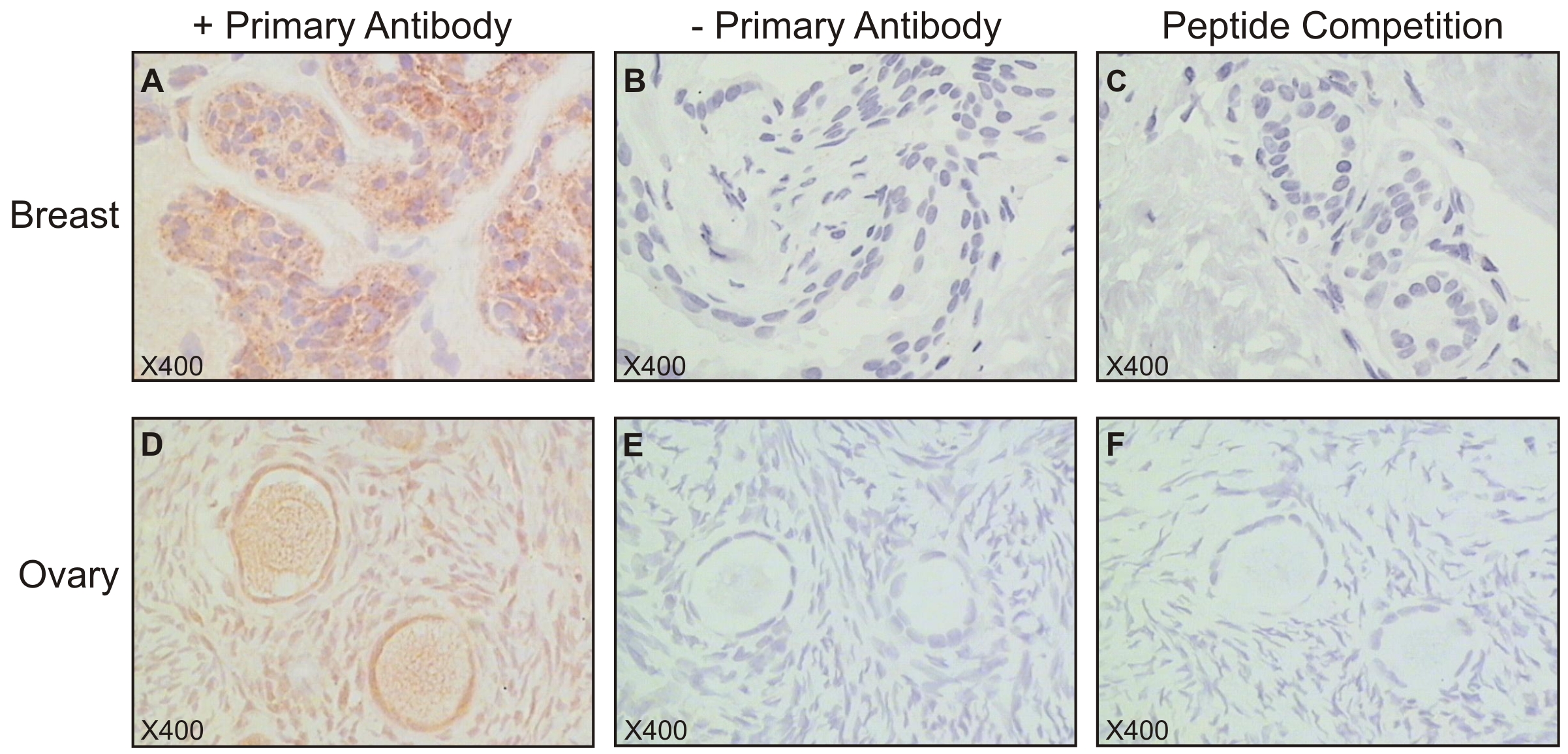

Supplement: Supplementary file 2 — KITL immunostaining in human breast and ovarian tissue. Human breast carcinoma (A-C) and human ovarian cortex tissue sections (D-F) used as controls for KL immunostaining. (A, D) Positive KITL staining. (B, E) Negative control which consisted of omission of the primary antibody. (C, F) Confirmation of KITL antibody specificity by incubation of the primary antibody overnight with its specific peptide prior to immunostaining. [file 13048_2015_159_MOESM2_ESM.jpeg]
